# Selections and Abstracts

**Published:** 1901-10

**Authors:** 


### SELECTIONS AND ABSTRACTS.

### Recent Epoch Making in Medicine.*

At a meeting of the British Pathological Society, of London, April 6th, 1875, the "Germ Theory" of disease was first formally introduced, the discussions being very animated and earnest, which also obtained at subsequent meetings. This conference was attended by distinguished medical men, some of whom were profoundly impressed by the arguments brought forward. The coexistence of bacteria and contagious disease was admitted, but Doctor Bastian, one of the most prominent speakers, contended that they are pathological products spontaneously generated in the body after it has been rendered disease by real contagion. The grouping of the ultimate particles of matter to form living organisms was considered by the speaker to be an operation as little requiring the action of antecedent life, as their grouping to form any of the less complex chemical compounds.

Prior to this, Henle (in 1840), after mature deliberation, collating and weighing of evidence, had arrived at the conclusion that the causes of infectious maladies are to be found in minute living organisms or fungi; hence he may be regarded as the true and original author of the "Germ Theory." He formulated opinions and investigated the subject with such thoroughness and ability that, in after years, Koch adopted precisely the same views. In 1862, Pasteur published a paper on the "Organized Corpuscle existing in the Atmosphere," in which was demonstrated that many of the floating particles are organized bodies, and that these, when planted in sterile infusions, yield abundant crops of microorganisms, evidencing that the source of life in the infusions was derived from the air. Listerism originated in 1875, and when Koch published his famous work on the *Wundinfectionskrankheiten* (traumatic infectious diseases), three years later, the Listerian

*Annual Oration by Samuel Bell, M.D., Michigan State Medical Society, Battle Creek, May, 1901.

theory took firm root, spreading slowly but surely to all departments of medicine and surgery.

From time to time, as the need was realized, men of genius have provided devices and instruments with a view to aiding in this work, and some of these have made possible subsequent discoveries. Among these may be mentioned the use of sterilized culture fluids as formulated by Pasteur; the introduction of solid culture media and the isolation methods of Koch; the use of the cotton plug by Schroeder and Van Dusch; the introduction of the anilin dyes by Weigert and, finally, the improvements made in the compound microscope.

It is interesting to note that after the discovery of the anthrax bacillus by Pollender and Davaine, in 1849, there was a prolonged period, during which no important discoveries of pathological organisms were made, but during this period important methods of *technique* were elaborated. This was again followed by a period during which important additions followed each other in rapid succession. In 1873, Obermeier discovered the spirillum that bears his name and is deemed the source of relapsing fever; Hansen, in 1879, announced the discovery of a bacillus in the cells of leperous nodules; and Neisser during the same year demonstrated the *gonococcus*; in 1880, the typhoid bacillus was first observed by Eberth, and subsequently and independently by Koch; the same year Pasteur published his work on "Chicken-cholera," and the *pneumococcus* was described by Sternberg; in 1882, Koch announced the *Bacillus tuberculosis*, which was soon followed by Pasteur's work on "Rouget du Porc," while Loëffler and Schutz reported the isolation of the bacillus of glanders; in 1884, the "comma bacillus" was announced by Koch as the probable source of cholera; about the same time Loëffler discovered the germ bearing his name conjointly with that of Klebs, and believed to be that of diphtheria, and before the end of the year the tetanus bacillus was demonstrated by Nicolaier; in 1892, Canon and Pfeiffer announced the bacillus of influenza; in 1894, Yersin and Kitasato independently isolated the germ of the bubonic plague, and Sanarelli discovered the *Bacillus icteroides*, supposed to be the source of yellow fever.

During the last quarter of a century the science of bacteriology has made triumphant strides, revolutionizing all preconceived ideas and theories respecting the ætiology, diagnosis and even the treatment of infectious diseases; among those upon which information has been of greatest value are tuberculosis, diphtheria, tetanus, bubonic plague, etc.

Up to 1875 there were few scientific men who accepted the germ theory, the great majority adhering to the doctrine of spontaneous generation, believing, with Billroth, that the presence of fungi, where decomposition was in progress, was an accidental result of universal distribution or (more conservatively) that their presence in putrid wounds was either due to spontaneous development or accidental and artificial introduction.

McFarlane was among the first of any prominence to accept the germ theory as applied to diphtheria. He says that all possible skepticism as to the specificity of bacilli was dispelled by an accidental infection that confined him to the house for three weeks during the busiest season of the year. Without having been exposed to any known contagion, and while experimenting in the laboratory with a virulent culture, the diphtheria bacillus was drawn into a pipette and accidentally entered his throat. As the result of this accident, two days later his throat was full of typical pseudo-membrane which contained Klebs-Loëffler bacilli.

Welsh, of Johns Hopkins, has perhaps furnished the most reliable as well as the most complete statistics of the results accomplished by the antitoxin treatment of diphtheria. Excluding every possible error of calculation, his report shows an apparent reduction of 55.8 per cent. in mortality. Another very important point made by this author illustrates the importance of early treatment, viz: The fatality in 1,115 cases of diphtheria, treated in the first three days of the disease, was about 8.5 per cent., as against 546 cases injected with antitoxin after the third day, with a death rate of 27.8 per cent. Thus was established the fact that early treatment is essential, and that after the toxin has set up destructive organic lesions in the various organs of the body, no amount of neutralization will restore the integrity of the parts; consequently, antitoxin fails to be of material benefit in the latter class of cases.

In 1884, Lusgarten devised a method for staining bacilli found in syphilitic tissue, which germs he assumed to be the cause of the disease. The most recent research on bacterium of syphilis is that of Van Niessen, who claims to have cultivated from the blood of a few cases, and by inoculation experiments obtained evidences of the specificity of the organism, by the production of abortion in pregnant rabbits; by the development of extra-genital primary lesions on the ears of the same in the form of nodes; and by the production of secondary ulcers, tumor-formations and irregular lesions. However, the researches of others, up to the present time, have not been satisfactorily confirmative, and consequently the specificity of this germ is not established.

Considering our increased possessions in the Far East, the importance of early recognition of the bubonic plague can be appreciated, especially when the United States Marine Hospital Service reports the introduction of this fell malady to the Western Hemisphere. Its appearance in Santos, Brazil, in October, 1899, marks an epoch in plague literature as furnishing the very first recorded instance of the disease in the New World. During November of the same year the malady was brought to New York by a British steamship, and late in December, 1899, it made its appearance in Honolulu; its advent in California is so recent that mere mention is sufficient.

This disease furnishes a striking illustration of the scientific advance of modern medicine, for it was not until 1894 that its true nature became positively known. All through the centuries, in all the countries, the subject had been enveloped in darkness, and there was a blind groping after facts, an unsuccessful search for cause, and the same ignorant struggle against its ravages, on the part of physicians, sanitarians and public officials alike, such as obtains to the history of cholera, a malady that now, fortunately, by the efforts of science, is robbed of its terrors. So, too, the cause of the plague, the mode of propagation and the measures essential to prevent its spread, are to-day matters of general scientific information. To Pasteur and Koch is indirectly due the credit of this discovery by establishing bacteriology as a science, though to a Japanese physician, Kitasato, and the French observer, Yersin, we are indebted for the discovery itself. The fact is now established that the plague is an infectious malady caused by a specific bacillus; and the anti-pest serum of Yersin and Roux, and the Haffkine prophylactic, have been tested with most gratifying results, the latter for the prevention of the plague, the former for its effects upon the bubonic poison whereby it is neutralized within the system. The French Commission that recently investigated the efficacy of the anti-pest serum in Portugal report that the mortality was but fourteen per cent. against seventy per cent. of fatalities where the serum was not employed.

In a recent lecture by Roux, a striking illustration was given of the efficacy of the Yersin serum:

> The Bombay manager of the local branch of the Credit Lyonnaise resided with his wife, children, and a numerous retinue of native servants, in a dwelling in an infected portion of the city. His little daughter was stricken with the pest in a virulent form; was treated with the serum and made a rapid and uneventful recovery. As a precautionary measure the whole family were subjected to innoculation and the same measure of treatment was offered to the native domestics. Those who accepted escaped infection, while all of the six who declined were stricken, five fatally. It seems that a more crucial test could not have been devised or a more triumphant vindication obtained.

The *British Medical Journal* gives the results accruing to the employment of Haffkine prophylactic in Bombay, which show a reduction in mortality of eighty to ninety per cent.

The work of the late Federal Commission in establishing the disputed fact that the plague existed in California was a signal triumph for science and marks an epoch worthy of a place in the archives of modern achievement.

In 1896, Widal and Grunbaum, working independently, discovered that when blood-serum from typhoid fever patients is added to cultures of the typhoid bacillus, a definite reactive phenomenon occurs; this is known as the "Widal reaction," and consists in complete cessation of the characteristic movement, and subsequent agglutination, of the typhoid bacilli. The test was applied to two hundred and thirty cases of typhoid, among troops engaged in the Spanish-American War, treated in the Medico-Chirurgical Hospital, and of this number two hundred and nineteen reacted positively, or 95.64 per cent. The statistics derived from Osler's wards in the Johns Hopkins Hospital by Block and Gwyn, up to November, 1898, evidence that the reaction was present in one hundred forty-four of a total of one hundred fifty-one cases. Statistics further developed the fact that the reaction failed in only 4.4 per cent. of cases out of a total of 2,393; and it is probable that even this small percentage would have been further reduced if the test, when negative at the first examination, had been repeated every day or two until convalescence was fully established. Without granting the precision of the method, it nevertheless may be assumed to be of great diagnostic importance.

Bacteriology is the outgrowth of the medicine and surgery of the past; and from being looked upon as merely incidental thereto it has become the dictator of the medicine of the present and future. Much valuable work has been done on the acute and chronic inflammatory diseases, also on the toxemias and bactericides.

Hæmatology, a comparatively new study, has become an adjunct to clinical diagnosis, but sufficient time not having supervened, the limits of its usefulness have not been fully determined; the evidences afforded, thus far, have been very disappointing sometimes—results accruing that were wholly unexpected, perhaps opposed to those sought—while again, on the other hand, shedding far more light than could have been anticipated. The number of maladies in which its value is apparent are less than a half-score, but that it proves a decided aid in many more is not to be gainsaid; it may provide the missing link in a chain of otherwise incomplete evidence. On the whole, hæmatology in its results is not inferior to examination of urine; both give definite results in a few diseases, and side lights in many obscure conditions, even if the process itself is negative and the former has one very decided advantage: It can be employed during the life of the patient, and like all methods of purely physical character, in all febrile maladies, and where there is any cause (such as insanity, stupidity or unconsciousness) preventing intelligent communication with patient, much light can thereby be obtained.

It is now conceded that *Anopheles*, a form of mosquito, may convey the parasite of malaria from man to man; even a *résumé* of the literature of the subject would consume so much time and space, I must, *per force*, be content with mentioning that important observations by original workers are now being made in the tropics that promise more practical information.

The most striking feature in this connection, however, is the (apparently) definite establishment that the cause of yellow fever is present in the blood of those attacked thereby, and that certain mosquitoes can inoculate healthy individuals; also that the disease is not transferable by fomites. This is regarded as a very important medical discovery, removing in large part the mystery obtaining to the ætiology of a malady that is not alone the scourge of some of the fairest portions of the globe, but renders certain districts thereof practically uninhabitable to civilized man.

Relative to the cause of cancer, Max Schuller, of Berlin, and Roswell Park, of Buffalo, (and the co-laborators of the latter at the State Hospital), have accomplished some excellent work. Park reports having been able, in some of the lower animals, to produce true adeno-carcinomas by inoculation with fluid from the peritoneal cavity of a man suffering with colloid cancer of the omentum. Schuller reports* having found in both carcinoma and sarcoma a golden-yellow body, a protozoön, that he presumes to be the primary cause of these growths; and a culture thereof, when injected into a rabbit, produced cancerous tumors, while other cultures revealed the organism in different stages of development. The results of experiments now in progress are awaited, both in Europe and America, with great interest.

Since the discovery of the Roëntgen rays, great advances have been made in the practical application of this mysterious form of energy. Somewhat reckless predictions, born of enthusiasm, have been indulged in, nevertheless they have proved of great diagnostic and therapeutic value, and time may be expected to establish more fully their scope and utility. At one time it was seriously feared that the prolonged exposure, deemed essential to successful observations, would limit their usefulness, but the improvements in *technique* that have recently accrued permit of excellent results being secured with more brief exposures. Now, with our improved

**Centralblatt fuer Bakteriologie*, 1900.

methods, a diagnosis is often possible, and with a precision that cannot be obtained in any other way. By means of the radiograph, foci of tubercular infection can be made manifest to the eye much earlier than to the ear; a unilateral or bi-lateral enlargement ot the heart, or any form of cardiac displacement, is readily discovered by the same means; emphysema, asthma, pleurisy, hydro-pneumothorax, pyopneumothorax, hydrothorax and pneumonia, are easily recognized and their limits defined; thoracic aneurysms are recognizable in their early stages; cavities which escaped detection by ausculation or percussion are revealed; and the presence of fluid within the pleura may be positively determined. Senn declared the X-rays as employed during the late war "fully answered all expectations," and added:

> "During the Spanish-American war the skiagraph enabled us to diagnose the existence or absence of fracture in a large number of doubtful cases in which we had to depend exclusively on this diagnostic resource In fractures in close proximities to joints, it has been of the greatest value in ascertaining whether or not the gunshot fracture extended into the joint. In the light of recent experience the X-ray has become an indispensable diagnostic resource to the military surgeon in active practice, and the suggestion that every chief surgeon of every army corps should be supplied with a portable apparatus, and an expert to use it, must be considerd a timely and urgent one."

Manifestly the limit of usefulness of this aid has not yet been determined. It may be noted, however, that the rays have rendered valuable aid in the treatment of diseases of the skin, more especially lupus vulgaris, lupus erythematosus, chronic eczema, vascular nævi, hyper-trichosis, favus, and sycosis; also in other pathological conditions of internal organs.

During the past ten years, phenomenal advancement has been made in the diagnosis and treatment of diseases of the stomach; the cyromele has been invented; the gastro-diaphane perfected; a perfect gastric electrode introduced, likewise the gastric bucket; X-ray pictures of stomach have been taken; a large number of lavage apparatus devised; the gastroscope made fairly practicable; and a number of operative procedures devised.

In considering the burning questions of the day it is requisite to include the bacterial toxins, sero-therapy, organo-therapy, auto-intoxication, and the relations of internal secretions to problems connected with the nervous system—that part of the human organism which, in the main, is responsible for the lofty position which man holds among animals. The last decade has given birth to unprecedented activity in connection with the progress in neurology. The results obtained have led to complete revolution in ideas concerning the elements of the nervous organs and their mechanical relations, and supplied a host of new methods of investigation in the prosecution of the study of the nervous system in health and disease. Entirely new avenues of research have been opened up, and problems heretofore thought beyond the reach of scientific inquiry seem now within human grasp. So numerous have been the methods of original research pursued, that space and time forbid their review. I shall merely mention briefly a few of the main achievements: Among the names which have shed new lustre on the subiect of neurology is that of Ramon y Cajal, whose connection with original work has been both brilliant and fruitful. If popular history can be relied upon, the story of this young scion of Spain is remarkable, especially from a medical standpoint. Developing in a country not remarkable for original research, he applied for a position as teacher of the microscope, and was refused; whereat, being ambitious, industrious and proud, he was keenly wounded. He then purchased a small library devoted to histology and microscopy, practically ostracised himself from society, and began his original work, paying special attention to *technique*, and as a result found himself, a decade later, famous. A brief inquiry into the contributions of Cajal cannot fail to reveal why, since 1888—and in all parts of the scientific world—his productions have attracted attention, and ultimately gained for him a professorship at Madrid, as well as notice and appreciation by international audiences. Among his original contributions are: "Demonstration of the Complete Independence of at Least the Majority of Nerve Elements"; "Appreciation of the Wide-spread Occurrence and Significance of the Lateral Branches of the Axis-Cylinder Processes," and "Demonstration of the Striking Uniformity in General Structure of the Majority of Nerve Elements in all Parts, Despite Minor Morphological Elements."

Since 1880, investigations of Golgi, His, Kölliker, Cajal and others have produced a complete revolution in ideas relative to the elements of which the nervous system is constructed, and also of the mode in which these elements are architecturally put together. The Golgi method of staining tissue is now recognized by the whole scientific world, and the pictures of nerve cells and their processes secured thereby (incomparably superior to anything hitherto obtained) are regarded in the light of a new discovery. Cajal with his incomparable genius made new applications of the Golgi method, which have attracted wide-spread attention, and anatomists everywhere (von Kölliker and others in Germany, van Gehuchten in Belgium, Retzius in Sweden, Schäfer and Andriezzen in Great Britain, Berkeley and Strong in America, and a host of others) set to work with the osmobichromate mixture and silver nitrate, and in a short time a new era was opened up, and information supplied regarding the reciprocal relations of nerve units in the various parts of the cerebro-spinal and sympathetic nervous systems. The connection of the axis-cylinder processes of the cells of the neutral horns with the axis-cylinder of the fibres of the motor roots of the spinal nerves, were first absolutely established by Weigert's methods coupled with the method of Gerloch.—This, in conjunction with improved *technique* in sectioning, has contributed greatly towards the investigations in neurology.

In 1891, Waldeyer brought out the doctrine of individuality of the nerve elements, or the "Neuron Concept," which may be briefly condensed as follows:

The nervous system, aside from its neurolgia, ependymal cells, blood vessels and lymphatics, consists of an enormous number of individual elements of neurons, each neuron in its entirety representing a single body or cell. The foundation for the neuron doctrine rests upon these facts:

The nervous system agrees with other parts of the body in being cellular.

The proof that in the embryo the nerve cells exist as independent units, many of which are capable of wandering for considerable distance from the origin.

The fact that the nutrition of the nerve cells is most easily explained from the standpoint of a doctrine which looks upon the nervous system as made up of units, which are not only anatomical but physiological.

Since this doctrine was advocated a large amount of work upon degeneration of nerve-fibre and cells has been done, especially by Marchi, which confirms the validity of the neuron doctrine, the latter being of value in enabling the histologist to follow the diseased nerve-fibre to its termination. The conception of the neuron has helped to facilitate the understanding of some diseases in showing that there is no cardinal distinction between gray and white matter; and it likewise served to unravel, in part at least, the mystery which formerly surrounded those diseases that involve, almost simultaneously, the various systems of white fibres and the gray matter. Proportionately with the growth of the neuron concept the value of systemic diseases is lessened.

Do what we may, we cannot separate mental diseases from organic affections of the spinal cord and, indeed, of many other organs of the body. The line of demarcation between the mental and physical conditions is so indistinct that, in many instances, one merges into the other. One of the foremost alienists of Europe latterly declared that psychiatry is on a level with the medical sciences of a hundred years ago, being based wholly upon clinical studies and not upon pathological anatomy. A few years since, Doctor Weir Mitchell, during his annual address to the American Medico-Psychological Association, indulged in secret criticism upon the lack of scientific work in hospitals for the insane, which aroused no trifling indignation. That abundant material for scientific work exists, both clinical and pathological, is undeniable, and that there has been marked advance, both in the character of the clinical work and in honest endeavor along ætiological lines, is conceded. That greater advancement has not been made cannot be attributed wholly in inertia on the part of those in charge, but is largely due (especially in relation to causation) to the well known fact that the morbid pathology of the brain is more complex than any other part of the human organism. The asylum reports, instead of being given over to stereotyped data, as formerly was the case, to-day are fast becoming storehouses of useful information regarding all that pertains to the care, cause, and treatment of the insane. To the practical psychiatrist the question of domiciliation does not overshadow every other desideratum, as in the past, and during the last decade the importance of early diagnosis, the accompanying pathological conditions, prompt separation from domestic surroundings, and skillful treatment (mental, moral and physical) have become questions of paramount interest. Closely trained observers, records of clinical facts, also systematic laboratory work, are now the rule rather than the exception in many institutions. Pleasant, cheerful rooms have taken the place of darkened cells; airy courts are provided, along with beautiful grounds and attractive architecture; the Kirkbride system has replaced the old quadrangular buildings, and the cottage pavilion, in some form, is fast superseding all others; finally, the specially trained and skilled nurse has been substituted for the ignorant (and sometimes careless) attendant.

The work of Meynert on the cerebral cortex, and the researches and experimental labors of Charcot, Flechsig, Wernicke *et al.* have done much to illumine conditions hithereto obscure and considered impervious; the labors of the New York Pathological Institute, under the leadership of van Gieson, have received the recognition and commendation of many of the original investigators of Europe as well as America, and the original contributions of Berkeley to the pathology of brain lesions have stimulated the study of psychiatry in insane hospitals everywhere. It is the spirit and honest endeavor on the part of those interested in the science of psychiatry (together with the increase of insanity over and above the increase in population) which makes possible a psychopathic hospital in Michigan in connection with institutions of learning, exactly as such are now established in connection with the older universities of Europe. At the present time there is no branch of medical science which offers so many interesting problems for solution; and though the past has been full of disappointments, the future is tull of hope.

We begin a new century under most encouraging auspices. That just closed will go down into history as one marvelous in scientific achievement, especially in many departments of medicine and surgery, and as marking the close of the career of dogmatic medicine; but there are still many important subjects that require careful and profound consideration. Fortunately, science recognizes no nationality; from Germany, Belgium, Sweden, Russia, Italy, France, Spain, Japan, South America, England, Canada and the United States, come reports of work that embody the spirit of scientific research to an eminent degree. What of the future? That more brilliant achievements are soon to follow, few can doubt. Serum therapy is yet in its infancy, and although one of the crowning triumphs of the Nineteenth Century, there are certainly great possibilities as regards its future scope and employment.—*Detroit Medical Journal.*

---

### MEDICAL TREATMENT OF THE DISEASES OF THE KIDNEY.*

To intelligently consider the therapy of renal diseases, it is necessary that they should be classified under *congestions, inflammations*, and *organic* change—bearing in mind always that one pathological condition may be, though not necessarily, consequent upon the other. The development of inflammatory change, and subsequent organic lesion, do not necessarily follow upon congestions. This last may not, of course, be a diseased condition *per se*, but form a part of some clinical picture, yet important in the sense of a grave complication, which may be the pivotal point in determining the result of the illness.

It seems to be a work of supererogation to say anything of the treatment of acute congestions; so plain are the indications that it is hard to go wrong. Yet the subject would not be complete without at least an outline of the most efficient means to be exhibited to bring prompt relief.

We find the patient with complete or almost complete suppression of urine, skin dry, some temperature, and if a sufficient time has elapsed, tendency to or pronounced uræmic coma. We must remember the vicarious office of the skin and bowels for relief of the kidneys, and by hot packs, administration of saline purges,

---

*Read by J. N. Upshur, M.D., Richmond, Va., President of the Tri-State Society of the Carolinas and Virginia, etc., before the Richmond Academy of Medicine and Surgery, September 10, 1901.

diaphoretics of pronounced action like pilocarpin, with turpentine stupes over the loins, relieve the system of harmful excrementitious matter, subsequently rousing the kidneys to active secretion by the exhibition of suitable diuretics, like flaxseed tea and lemon, hot lemonade, acetate of potash, etc. Be careful always to select such agents as will be at least irritant to the kidneys, and therefore less likely to enhance the trouble by producing irritation, and increasing rather than diminishing the congestion. Care should be taken in the subsequent treatment that the kidneys are continuously well flushed by lithia in some form; by regulation of diet, so that an unusual amount of excretion be not required as the result of gastric derangement. The diet should be light but nutritive. Alcohol in all forms should be forbidden, and the subject of the attack is better off without either tea or coffee. The body, especially over the loins and abdomen, should be protected from sudden changes of temperature by soft, warm flannels.

When passive congestions are present, it is absolutely requisite to intelligently treat the condition that careful and analytical discrimination should be made as to the cause, in order that it may be removed. To attempt to stimulate the kidneys under these conditions is like whipping a foundered horse; it only adds to the gravity of the trouble and does harm instead of good. These passive congestions have usually as their prime cause feeble heart action, whatever may be the condition back of it and acting as a depressant; low fever, septicæmias, miocarditis in some form, failure of compensation in valvular lesion, animal poisons, such as the infection of diphtheria, or other exanthems, or super-saturation with drugs, as digitalis, when it has been administered too long and the third stage of the physiological action has been established.

To brace up the heart and make it perform its function as nearly in a normal manner as possible is the indication for relief; and the selection of the agent requires a just and intelligent discrimination; care—great care—must be taken that no agent is selected which can directly or indirectly be depressant. The salicylates in every form, whether as a drug or as a constituent of those proprietary agents into which it enters as the most prominent factor, as diuretin, should not be considered for an instant, as they are positively contra-indicated. Nitro-glycerin, erroneously spoken of and considered as a heart tonic, can be only fruitful of evil, because it is purely and simply a cardiac depressant through its paralysant action on the vaso-motor nervous system. Alcohol moderately, but caffein, strychnine, strophanthus, are agents upon which we may rely, encouraging the drinking of an abundance of pure water and paying careful attention to nutrition.

In acute inflammation we have a different pathologic condition to deal with. The kidney is acutely enlarged and needs depletion by such agents as will make the blood-vessels unload themselves. Counter-irritation to the loins with turpentine stupes, free derivative action from the kidneys through the skin by exhibition of pilocarpin, and other active diaphoretics, including the hot pack, from the bowels by full and repeated doses of Epsom salts, stimulation of the kidneys to a freer secretion by such agents as nitro-glycerine, given in doses of 1-50 to 1-20 grain every six, four or even two hours if necessary. It acts in this condition by dilating the tense blood vessels and giving more room for the forwarding the current of blood previously domed up and threatening development of a hyperplasia in the organ, and relieving the heart tension by diminished resistence in front.

In these conditions, too, especially when a sequel of scarlet fever with pronounced dropsy, infusion of digitalis, serves a good part, pushed to a physiological point, where it produces diminished tension, causing a very free flow of urine. It is, I know, a very nice distinction to make of simple inflammation of the kidneys and an acute parenchymatous nephritis, and possibly it is only one of degree, but the duration of the attack can be understood to discriminate between the two. In parenchymatous nephritis, tubular, vascular, and interstitial tissues are all involved, and the condition is more grave, and much more apt to be followed by serious degenerative organic change, requires, too, more active and longer continued treatment. Acute parenchymatous nephritis does not infrequently terminate in complete recovery under appropriate treatment, but is also a very common thing for it to run into a chronic condition; analysis, chemical and microscopic, showing abundance of albumen—persistent also—and kidney debris and blood and epithelia and dark granular casts, blood corpuscles and free epithelium and fatty cells in the urine.

In the treatment of this form of trouble, rest in bed, quiet and warmth, are the indications; the exhibition of such agents as will combat the suppression of urine, with all the ills that follow it. The patient becomes enormously dropsical very soon, and the swelling and tension are sources of great discomfort. Agents, therefore, should be selected which not only stimulate the kidneys to increased action, but promote absorption. Infusion of digitalis is highly recommended at the outset, acetate and bitartrate of potash, lemonade, etc., being added just as soon as the digitalis shows its physiological effect on the pulse. (This is the second stage of its action, diminished tension and increased frequency.) I prefer other agents where there is vascular tension, with hard, firm pulse. I know of no agent which relieves the condition more promptly and efficiently and causes as abundant flow of urine as nitro-glycerine; coincidently may be given simple diuretics, abundance of lithia water, lemonade, acetate of potash, etc.; saline purgatives, as well as others of pronounced hydragogue action, are very valuable and tend to increase rather than depress the strength of the patient. They exert also a derivative influence from the brain, and, eliminating the retained effete material, tend to counteract the uræmic coma, which always threatens. It also diminishes the tendency to convulsions. Vomiting or nausea is often a distressing symptom, and is to be relieved by such agents as are sedative and soothing to the mucous coat of the stomach. Distressing itching of the skin, which is sometimes present, may be relieved by rubbing freely with menthol in almond oil.

When acute mania, delusional insanity, are present, resort should be had to bromides, especially bromide of potash, because the potash element tends to the kidneys for elimination and with increased secretion. Morphia should be avoided; indeed, all preparations of opium, because of their pronounced interference with secretion, though sometimes the urgency of the symptoms to be controlled is so great that, much as we may deprecate it, opium, or some one of its alkaloids, has to be exhibited for temporary relief. I have found hyoscyamin, in dose of gr. 1-100 hypodermically, very useful in procuring sleep and quieting nervous irritation. To the nurse it manifests a sleep very noisy in the beginning, but the patient is unconscious of it and awakens much refreshed. In large measure, the treatment above outlined in the acute is indicated in the chronic form of parenchymatous nephritis, but some modification will usually be found to be necessary.

It must be remembered that many cases of the chronic form come on insidiously without having been preceded by the acute. Poverty of blood and general systematic depression usually are prominent; and a sustaining treatment, both by concentrated nutritious diet and appropriate tonics in the form of Basham's mixture, manganese, arsenic, and strychnine, are indicated; but care should be taken not to exhibit the iron in too large doses, as it will lock up the secretions and defeat the object for which it is administered.

Careful attention should be paid to diet. The less meat taken the better, but an *abundance* of good sweet milk or buttermilk, diluted with Vichy water, is desirable; succulent vegetables may be allowed; tea, coffee and alcohol should be prohibited. Hot pack may be administered daily for its effect on the skin and depurative effect on the blood. The time comes, however, when all means fail, and we can only endeavor, alas, often succeeding but poorly, to make the patient comfortable.

What has been said fully of the treatment of chronic parencymatous nephritis applies equally to the treatment of the interstitial form; it must be largely symptomatic. I would emphasize the contra-indication for the exhibition of mercury; the tendency to ptyalism is very strong, and when it does occur it is very intractable, going on to a condition of aggravated cancrum oris. In the last stages, when distress is extreme, resort must be had to some form of anodyne, potent enough to give relief from suffering. I have been forced repeatedly to resort to some form of morphine and atropine. I have had very good results from the exhibition of hyoscyamin, gr. 1-100, given at bedtime hypodermically.

The treatment of *lardaceous* kidney is largely anti-syphilitic, or by the removal of a limb in which exists profuse chronic suppuration or suppurative pyelitis, or pyelonephritis, perinephritis, or perinephritic abscess. The treatment belongs so eminently to the domain of the surgeon that I pass it by as not being within the scope of this paper.

*Tubercular* kidney requires general upbuilding of the system, just as when tubercle exists in any other organ or region.

*Kidney colic.*—The treatment is palliative at the time of the paroxysm, and dietetic during the interval, the form of the diet depending on the composition of the stone or sand. If uric acid dominate, the exhibition of alkalies is indicated, especially alkaline mineral waters. A limit should also be put to the amount of notrogenous diet. If the tendency be phosphatic, benzoic or boric acid has the reputation of being efficient, but these are not well borne by the stomach. The mineral acids, especially dilute muriatic, will be found useful. In the matter of diet, meats and milk are indicated, and a diminution of the vegetable element because of their tendency to alkalinize the urine. The urine should be carefully watched for phosphatic deposits.

The only medical treatment in floating kidney is in the direction of upbuilding of the general health, resort to surgical procedure being required in many cases.

Hemorrhage from the kidneys is to be combatted by removing the cause, whatever that may be. It is sometimes due to sharp crystals of oxalate of lime passing through the kidneys; and I have found, when this is the cause, most decided benefit from the exhibition of the tincture of guicum, teaspoonful every two, four or six hours.

Finally, so common is cardiac involvement present with kidney lesion that careful discrimination should be exercised to determine which is the primary condition, and the remedies must be directed accordingly.—*Va. Med. Semi-Monthly.*

---

### ATTEMPTS ON THE LIVES OF PREVIOUS PRESIDENTS.

*Medical History of Garfield's Assassination.*—On July 2, 1881, at 9:20 a. m., President Garfield was shot by Charles J. Guiteau, a man who had been disappointed in seeking for an office. The scene of the assassination was the station of the Baltimore and

Potomac Railway in Washington. The weapon employed was a British bulldog revolver of 0.44 calibre, with 20 grains of powder and a bullet weighing 200 grammes. The range was eight feet. The assassin fired two shots, the second of which took effect. At the first shot the President turned, and the second shot entered opposite the tenth intercostal space, about four inches from the median line, on the right side. As the bullet struck him, the President fell on his knees and then on his right side, vomiting as he fell.

Almost immediately symptoms of cardiac failure set in, and when, four minutes after the shots had been fired, Dr. Smith Townsend came in, he found the President in a state approaching syncope. He administered brandy and aromatic spirits of ammonia, and made a hasty examination of the injuries. The vomiting continued from time to time, and after a second dose of stimulants the patient rallied. In a few minutes Dr. Purvis and Dr. Bliss arrived, and the patient was moved to an upper room of the station, where he rallied from the first shot and was prepared for removal to the White House. He complained of pricking sensations in the right leg and foot.

On the arrival at the White House an effort was made to ascertain the nature of the injury, and the President was fully conscious and cheerful, but his pulse was weak and rapid. The entrance of the wound was found as above stated, and on palpation the eleventh rib was found fractured. The probe passed downward and forward. The wound was dressed antiseptically and morphine given for the pain. At 4 in the afternoon the pulse was 132, the temperature subnormal, 96.8° F., and there were evidences of internal hemorrhage. At 11:20 p. m., the reaction from the shock began and the condition improved gradually. On the morning of the 3rd the temperature and respiration were normal, and the pulse was 115, the patient resting comfortably. At 2 p. m., there were vomiting, slight tympanitis, and marked rigidity of the abdominal muscles, but no pain and no rise of temperature beyond 100°.

On the 5th, Dr. D. Hayes Agnew, of Philadelphia, and Dr. Frank H. Hamilton, of New York, were called in consultation.

As it was impossible to find the direction of the ball, no interference was advised. The progress from this point seemed favorable. The patient took liquid nourishment, the fluctuations in pulse, temperature and respiration were not important, but he complained of pain in his feet. On the 23rd the patient had a chill, followed by a pulse of 120 and a temperature of 104°. Two days previously a pus sac had been discovered extending under the skin and subcutaneous tissue below the twelfth rib, under the latissimus dorsi, and it was evacuated by gentle pressure. This sac was considered to be superficial, and not the cause of the symptoms; a free incision was made and small fragments of the broken rib were removed. On July 26th the opening between the fractured ends of the eleventh rib was enlarged and the detached removed. Now the condition remained more or less uniform until the 6th of August, when a slight febrile movement was observed. A soft catheter was passed into the wound and reached the crest of the ilium. The wound was washed out through the catheter with potassium permanganate. On the 8th it was decided to drain at the dependent part of the tract, and this was done, the necessary opening being made under ether. The operation was followed by comparative improvement. On the 14th there was vomiting and no food could be retained, so that rectal feeding had to be used till the 17th. On the 18th a swelling of the right parotid appeared and developed into an abscess; this was accompanied by facial paralysis, which later slightly improved. The channel of the wound was kept clean by irrigations with potassium permanganate and carbolic acid. At about this time a number of small pustules appeared on various parts of the body, probably as the result of sepsis. On August 26th there was a discharge of the parotid abscess into the ear and mouth, and the inflammation extended to the pharynx, larynx, trachea, and bronchi. Acute catarrhal bronchitis developed and there was some hypostatic condition due to the decubitus. The abscess was evacuated and the condition improved.

The President was now removed to the seashore at Elberton, N. J., where he gradually improved until September 15th, when there developed a limited broncho-pneumonia. On the 17th he had a severe chill, followed by a sharp rise of temperature. Mental disturbances were now noticed, and there was a pain over the anterior mediastinum which recurred every six to twelve hours. This pain was caused by the rupture of an aneurysmal sac which dissected progressively, at irregular intervals, the surrounding cellular tissues until it burst into the peritoneum. On the 18th there was another chill and the pains continued at intervals. The fever fluctuated considerably during these last days.

On the morning of the 19th the pulse was 106, feeble, the temperature 108.8° F. (?), and there was another chill. The day was passed in comfort, but the alarming symptoms of the morning did not abate and the President died at 10:10 p. m.

The post-mortem showed the following conditions: The ball had entered opposite the tenth intercostal space about four inches to the right of the median line. It went forward and downward, inclining a little from right to left. It impinged upon the eleventh rib and produced a comminuted fracture thereof. Thence the bullet was deflected to the left, and pierced the eleventh external intercostal muscle and the subpleural portion of the diaphragm, just above the right ligamentum arcuatum. It then went through the circumrenal tissue between the right kidney and twelfth rib, and pierced the attachment of the psoas at the first lumbar vertebra. It went through the body of the first vertebra from right to left, emerged at the left of the spine and pierced the left psoas, entered a plane of adipose tissue between the left kidney and the pancreas, crossed the posterior surface of the pancreas, wounding the splenic artery in transit, and was lodged (encysted) in the external third of the pancreas. In addition to the fractures of the eleventh and twelfth ribs and of the body of the first lumbar vertebra, there had therefore been a wound of the splenic artery.

The pus burrowing down from the ribs formed a superficial abscess and then burrowed downward and forward in the direction where the ball was supposed to have gone. The pain in the feet and inguinal region is explained by the vertebral injury, and the wound of the splenic artery was followed by a traumatic dissecting aneurysm which caused death when it emptied into the peritoneum.

It was extremely unfortunate that the complicating fractures and ensuing caries and suppuration should have misled the surgeons as they did. Had the correct diagnosis of the bullet's course been made, the prognosis might not have been so unfavorable.

*Medical History of Lincoln's Assassination.*—The medical history of Lincoln's case was brief, for he lived but a few hours after the fatal bullet had entered the brain. On the evening of April 14, 1865, the President, accompanied by his wife, Major Rathbone, and Miss Harris, attended the performance of "Our American Cousin" in Ford's Theater, Washington. The Presidential party occupied a box near the stage. At about 10:30 p. m., John Wilkes Booth, an actor, jumped into the box from the stage and fired at the President's head from behind, holding his weapon, a "common single-barreled pistol," at close range. The bullet entered the cranial cavity through the occipital bone, a short distance above and behind the left temporal, and passed through the cerebral tissue forward toward the frontal lobe. There is said to have been little hemorrhage immediately after the injury, though a newspaper stated next morning that the box was found bespattered with blood. Brain substance was, however, oozing out of the wound from the first. The President became unconscious immediately, and did not regain consciousness until the end. He was removed, as soon as the confusion would permit, to a private house opposite the theater and the Surgeon-General was sent for. The respiration was labored, the pulse 44 and feeble, the eyes were closed, the left pupil was contracted, the right widely dilated, and there was in both pupils total insensibility to light. At the theater some one called for stimulants, and an attempt was made to make him swallow some brandy, but this was impossible. At 11:30 p. m. twitching of the facial muscles on the left side set in and continued for fifteen to twenty minutes. Soon after the patient was placed in bed, the wound began to discharge blood and brain tissue, and this discharge continued till 5:30 p. m. It was noticed that so long as the discharge continued, the respiration and pulse were fairly good, but no sooner had it ceased than there were changes for the worse in both pulse and respiration. The patient's head was held so as to facilitate the flow from the wound. At 2 p. m. the wound was probed with a silver probe, and about three inches from the entrance it met an obstruction in the shape of a piece of the occipital bone. Two inches farther the bullet was felt, and, on passing it, the sound came upon fragments of the orbital plate of the left orbit. No further probing was attempted. The bedside notes taken by Dr. Abbott (*New York Herald*, Sunday, April 16, 1865) show no record of temperature, but the pulse and respiration and the other principal symptoms were recorded every five minutes. At 12:30 p. m. ecchymosis appeared in both conjunctiva, and at 12:55 the patient became restless and struggled with his arms. At 1:3C he became more quiet, and continued so until the respiration became regular and he fell asleep. At 6 a. m. the pulse began to fail alarmingly. At 7 symptoms of dissolution were noted, and at twenty-one minutes and fifty seconds past seven, Lincoln breathed his last, his heart continuing to beat for a few seconds longer. At the autopsy the bullet was extracted from the frontal lobe.

From the first moment there had been practically no hope, and at 1 a. m. the newspapers printed dispatches from Washington saying the President would not live through the night.—*New York Medical Journal.*

---

### SUGGESTIONS BEARING UPON THE DIAGNOSIS AND TREATMENT OF FRACTURES AT THE LOWER END OF THE HUMERUS.*

A somewhat extensive experience in the management of fractures at the lower end of the humerus, together with frequent opportunities afforded me for the inspection of cases in the hands of capable, painstaking colleagues, has left me with the conviction that the results obtained by the accredited methods of treatment are far from satisfactory.

Every general surgeon has upon his list one or more cases illustrative of this point. Too often pain, deformity, and limited function cripple the patient and humiliate the attendant. It matters little that the patient has been forewarned as to what have been regarded as inevitably disastrous consequences of his injury, nor does the approval of able counsel as to the line of treatment adopted free his mind from the impression that the result ought to

*By Thos. W. Huntington, M. D., San Francisco.

have been more satisfactory. With the facilties now at hand is it possible to establish and maintain a higher standard of excellence in the treatment of elbow-joint fractures? The time has come when surgeons should answer this question definitely. From our large hospitals, wherein the pace is set so far as surgical achievement is concerned, there should issue an edict setting forth the rules of practice governing these cases, thereby approving or condemning methods which now prevail.

Fractures in this locality suggest a multiplicity of lesions, whose pathology and individual peculiarities have been adequately described, demonstrated, and illustrated. Their limitation is between the supra-condylar line and any imaginable communication of the lower end of humerus. Their thorough understanding depends upon an accurate determination of three points: 1. The number, relation, and direction of fracture lines. 2. The extent and character of displacement or dislodgment of fragments. 3. The relation of the fragments to the ulna and radius.

It is absolutely essential, in considering these points, that appeal be had to the radiograph for a satisfactory interpretation of existing symptoms. No longer can the visual and tactile senses be implicitly relied upon for every link in the chain of evidence. It is true that the determination of the existence of a fracture, together with its general features, is a comparatively easy task. To predicate its exact limitations, to draw an accurate clinical picture, to identify each individual fragment and give to it its true value in relation to prospective deformity or impairment of function, is most difficult of accomplishment. Surgeons will continue to inspect and palpate, to compare and manipulate. The three bony prominences of the elbow will be appealed to for such evidence as they may afford. The condyles will be scrutinized for mobility, crepitation, or faulty relations, but in most cases the evidences will be incomplete and altogether inadequate until one or more shadow pictures have been carefully inspected and interpreted. Aside from diagnostic measures, successful treatment of these fractures depends upon three factors: 1. Perfect readjustment of fragments. 2. Permanent maintenance of normal relations. 3. As early passive movement as is consonant with safety.

It is not my intention to advocate any conventional plan in dealing with this most perplexing problem. Flexion or extension of the forearm as a universal law are questions that interest me very little, if at all. My contention is that the surgeon must be certain of his initial adjustment, and as certain that proper relations are maintained throughout the progress of the case. This point seems not to have received the attention it clearly merits, and to this fact can be attributed many disastrous results. What right has the surgeon to assume that faulty relations will not be re-established within a few hours or days subsequent to the first dressing? And in the presence of such a state of affairs, can he be justified in deferring corrective measures until the integrity of the joint is hopelessly impaired? On the contrary, the more rational plan is to make the initial adjustment as perfectly as may be in the presence of the diagnostic radiograph, with the patient under an anesthetic. The position of the forearm will be negotiated so as to conform to the requirements of the case. A light dressing will then be adjusted, eliminating all thought of the maintenance of the fragments by direct pressure. For this purpose, plaster of Paris has advantages superior to any formal splint. The corrective position should be carefully maintained by an intelligent person until the cast has become self-supporting. Within twenty-four or forty-eight hours the initial work should be checked up by a second radiograph. If there still remains a fault in adjustment or alignment, a second or a third attempt should be made for its correction.

I am fully aware of the fact that the general practitioner whose life is spent in the isolated communities of the interior cannot be expected to maintain nor is he often within easy reach of an X-ray laboratory. That must be conceded to be his misfortune and not his fault. But it must be remembered that the work done in this line is coming to be regarded as constituting a specialty, and it is to be hoped that X-ray laboratories conducted by photographers or other competent workers will be established with profit in most of our interior towns.

The matter of early resort to passive movement may be dealt with in a few words. Assuming that correct positions have been maintained for five or six days, the joint surfaces will admit of considerable play without disarrangement of fragments or unwarrantable pain. In pursuance of this plan I have usually removed the splint on the fifth day. Movement is affected through a small arc. If the forearm occupy the semi-flexed position, I carry it up toward a right angle through an arc of ten or fifteen degrees. If this has been done withont appreciable violence, fixation in the new position is secured as before. In this manner the effort is renewed, and the position changed every second or third day, with successive increase of motion. There are occasional cases which seem wholly incorrigible so far as manipulative measures are concerned. Here the possibility of an unfortunate result under ordinary measures becomes an assured fact. Under these circumstances I should have no hesitation in converting a closed to an open fracture under proper precautions, ascertain the discrepancy, and wire or suture the obdurate fragments. The thought to be borne in mind in this connection is that if such a step be taken at all it must not be delayed. The greatest possible benefit to be derived from operative treatment can only be realized before efforts at repair have progressed to any appreciable extent.

It was not my intention at this time to enter upon any detailed description of special fractures or their sequels, but I am certain that you will be interested in one phase of the subject, which seems not to have been generally understood—"gunstock deformity," or, according to the more recent nomenclature, "cubitus varus," a condition or deformity resulting from this class of fractures more often than is generally supposed. About twenty years ago Dr. Oscar H. Allis, of Philadelphia, called attention to this deformity, and subsequent authorities have dwelt upon it at considerable length. It consists in a permanent adduction of the forearm resulting in obliteration of the obtuse angle, which normally is made by the axis of the humerus on the one side and the radius on the other. This has come to be known as the carrying angle, enabling a weight to be suspended from the extended arm without striking the leg of the bearer. This condition usually exists without interference with rotation or flexion of the forearm. It has generally been ascribed to a sliding upward of the internal or sliding downward of the external condyle. Very recently, in a most elaborate article in

4

the *Annals of Surgery* for September, 1900, Stinson has demonstrated that this deformity is associated with transverse supracondylar fractures of the humerus. In one of my own cases, occurring in a child of seven years, this condition occurred in mild form after a complete separation at the epiphyseal junction. Logically it would seem that the obliteration of the carrying angle may be attributable to any of the foregoing conditions, that is, fracture of the internal or external condyle, fracture above the condyles, or separation of the epiphysis.—*Occidental Medical Times.*

---

### HISTORICAL NOTES OF PRESIDENT MCKINLEY'S CASE.

The President was shot at 4 p. m. Friday, September 6. The revolver used was of .32 caliber. At 5:24 he was operated upon at the Exposition Emergency Hospital. The surgeons present were Dr. Matthew D. Mann, Professor of Obstetrics and Gynecology, University of Buffalo; Dr. Herman Mynter, Professor of Operative Surgery, University of Buffalo; Dr. John Parmenter, Professor of Anatomy and Clinical Surgery, University of Buffalo; Dr. E. Wallace Lee, of St. Louis, Mo., who happened to be on the Exposition grounds; Dr. Eugene Wasdin, of the Marine Hospital Service, and Dr. Presley M. Rixey, physician to the President.

The operation was performed by Dr. Mann; first assistant, Dr. Herman Mynter; second assistant, Dr. John Parmenter; third assistant, Dr. Lee. Dr. Eugene Wasdin gave the anesthetic, which was ether. The operation lasted forty-five minutes. Dr. Rixey arrived at the latter part of the operation. There were two wounds, one about the center of the sternum, the other two and one-half inches below the free costal border on the left side and in the semilunar space. It was this latter wound which necessitated the speedy operation. A five-inch incision was made and the course of the bullet followed up. It was found to have penetrated the anterior wall of the stomach. The opening was small and was sutured with silk. After closing this a search was made of the intestines; the stomach was lifted from its position and a larger perforation was found in its posterior wall. It was over an inch in diameter, jagged and irregular. This wound was sutured with black silk also. Continuous catgut was used for the fascia. The skin was coaptated with six through-and-through silkworm stitches, and interrupted catgut was passed between the silkworm gut. The abdominal cavity was thoroughly irrigated with normal salt solution. There was very little food in the stomach at the time of the shooting; because of the dexterity of the operators and this happy incident no foreign matter entered the peritoneal cavity from the stomach. The wound was closed with catgut, without drainage, and bandages were applied. The bandages were removed at 8:30 a. m. Saturday and showed but little staining.

Before anesthesia had passed off the President was conveyed by ambulance to the residence of Mr. John G. Milburn, president of the Pan-American Exposition, the Emergency Hospital being for temporary use only.

Dr. Rixey and one of the surgeons were with the President each night, and consultations of all the surgeons were held three times a day. Dr. Roswell Park was at Niagara Falls and came on a special train and assisted during the latter part of the operation. Dr. Clarles McBurney, New York, was called in consultation on Saturday.

Other physicians who were called into the case later were Dr. Charles G. Stockton, of Buffalo, and Dr. E. G. Janeway, of New York.

The female nurses in attendance on the President were Miss Helen Mohan, a graduate of the Buffalo General Hospital Training School; Miss Conley, a graduate of the same school and lately clinic nurse at the German Hospital; Miss Hunter, nurse of Mrs. McKinley, and Miss Grace M. McCullough, of Baltimore. The male nurses were selected from the detachment of the United States Army Hospital Corps on duty at the Exposition. They were Acting Hospital Steward Palmer A. Eliot and Privates Ernest Vollmeyer and John Hodgins.

Barring the rapid pulse there were absolutely no untoward signs or symptoms for several days. During Tuesday night two stitches were removed from the external wound on account of the perceptible irritation. The kidneys and bowels began to functionate normally. Until early Tuesday morning the President received stimulants only hypodermically, but at that time nutrient enemata of eggs and whisky were begun. In the afternoon beef juice was given by the mouth. Sedatives were not necessary, as the patient slept fairly well.

The first bulletin after the operation showed the pulse to be 124 and the temperature 102°. The temperature (rectal) did not run much above 102° at any time, being near 100° the greater part of the time, but the pulse continued frequent, varying from 104 to 120 and remaining about 120 mostly. Tincture of digitalis, strychnin and adrenalin were given from time to time. Thursday afternoon the pulse mounted to 126 and the President showed signs of weakness. During the day he had been given a small bit of toasted bread about an inch and a half square, mainly for the purpose of cleaning out his mouth.

With the approach of night the patient's condition grew somewhat worse, and Friday morning about 1 o'clock he was in a precarious state, the circulation being defective. Stimulants were given freely and he was considerably improved within a few hours.

Blood counts made by Dr. Eugene Wasdin demonstrated the absence of leucocytosis. The differential count of leucocytes showed no departure from normal and the usual number of red cells was present. Thus the evidence derived from the blood-counts indicated the absence of any sepsis.

As the day passed he again grew weaker and lapsed into unconsciousness at about 8 o'clock in the evening. Oxygen was administered without avail. He died at 2:15 Saturday morning.

### THE AUTOPSY.

The following report of the antopsy upon the remains of President McKinley was issued Saturday afternoon :

The bullet which struck over the breastbone did not pass through the skin and did little harm. The other bullet passed through both walls of the stomach near its border. Both holes were found to be perfectly closed by the stitches, but the tissue around each hole had become gangrenous. After passing through the stomach the bullet passed into the back walls of the abdomen, hitting and tearing the upper end of the kidney. This portion of the bullet track was also gangrenous, the gangrene involving the pancreas. The bullet has not yet been found.

There was no sign of peritonitis or disease of other organs. The heart walls were very thin. There was no evidence of any attempt at repiar on the part of nature and death resulted from the gangrene which affected the stomach around the bullet wounds as well as the tissues around the further course of the bullet. Death was unavoidable by any surgical or medical treatment and was the direct result of the bullet wound.

(Signed)

HERMAN G. MATZINGER, M.D.,
HARVEY D. GAYLORD, M.D.,
P. M. RIXEY, M.D.,
MATTHEW D. MANN, M.D.,
HERMAN MYNTER, M.D.,
ROSWELL PARK, M.D.,
EUGENE WASDIN, M.D.,
CHARLES G. STOCKTON, M.D.,
EDWARD G. JANEWAY, M.D.,
W. W. JOHNSON, M.D.,
W. P. KENDALL,
Surgeon U. S. Army;
CHARLES CARY, M.D.,
EDWARD L. MUNSON,
Asst. Surgeon U. S. Army;
HERMANUS L. BAER, M.D.

—*St. Louis Medical Review.*

---

### THE TREATMENT OF FOREIGN BODIES IN THE EYE.

Dr. E. S. Thomson (*Post-Graduate*), in the course of an article on wounds and injuries of the eye, delivered before the New York Post-Graduate Clinical Society, gives as follows the treatment of foreign bodies within the eyeball:

The treatment of foreign bodies in the globe, if the wound of entrance is not in the ciliary region, should be the early removal of the foreign body. The Roentgen rays may sometimes be of advantage in locating the foreign body, but are not so generally useful as in other branches of surgery. If the foreign body is of stone or glass, the attempt should be made to remove it with forceps, even at the risk of severe reaction. It it is of metal which can be magnetized, the removal by the electro-magnet should be attempted. Wherever possible the wound of entrance should be used, but if we can by an incision get nearer to the foreign body, a new incision should be made, as the power of the magnet decreases enormously with the distance from the foreign body. The size of the body is also very important, for as the attraction lies between the molecules of the foreign body and the magnet, the larger the foreign body the greater the attraction. In order to exert a strong force upon very small particles, the large Haab magnet has been devised. This is a very valuable instrument, but is falling into disfavor in Europe on account of the violence of its action. Where the piece of metal is even as large as an average of two mm. in its three dimensions, the Haab magnet will tear it violently from the eye, often doing severe damage. It need hardly be said that a smaller magnet should be first used, and we should only resort to the Haab magnet if the smaller one fails.

If the foreign body is small, and lies back at the posterior pole of the eye, we may try several times before we get any response. This is not due to the necessity of magnetizing the particle, as has been stated, for the attraction is exerted instantly, but to the fact that the attraction is so feeble, on account of the size and distance of the particle from the magnet, that the first efforts only succeed, making the particle move slowly forward through the vitreous. As soon as it comes near enough to be within the sphere of attraction, it moves quickly forward out of the eye and flies to the tip of the magnet.

The method of procedure should be as follows: The eye being cocainized and a proper opening made, the patient should be gradually approached to the magnet until the tip touches the globe near the wound. This should be done five or six times and plenty of time allowed for the particle to come forward. If the particle strikes the ciliary body or iris, or pulls any exudates, we have sudden pain, which is valuable from a diagnostic standpoint. If the removal be successful, and the resulting iridocyclitis is not severe, we have little trouble. If the foreign body remains in the eye near the ciliary region, enucleation should be performed. If the situation is not known, we may temporize and try to save the eye, but at the first symptoms of irritation in the fellow eye the wounded eye should be enucleated. It must never be forgotten that an eye containing a foreign body is a serious menace to its fellow, and if the vision is very much damaged, it should be sacrificed without delay.

Dr. E. Villiers Appleby (*Journal American Medical Association*, June 22), in an illustrated article on magnetic foreign bodies in the eye, speaks of the use of the Roentgen rays for localization of the foreign body. He says that in order to obtain good results in X-ray work, the head and eye of the patient must be kept motionless during exposure. This is best accomplished by having the patient lie upon a table designed for that purpose. The eye should be kept closed. Knowing that the rays travel in straight lines, we endeavor to place the tube and plate in such a position that we get a bi-temporal skiagraph, and also one taken in a fronto-occipital direction.

Before making an exposure in the bi-temporal direction, four pieces of fuse wire, each 6 mm. in length, are placed, and held in position by means of ordinary court-plaster, over the temporal region of the eye, so that the enclosure represented between their inner ends corresponds to an area a little larger than the lateral area of the eye. These pieces of fuse wire show distinctly on the plate after development.

If there be upon the plate more than the four regular outlines of the wire, we know we have a foreign body, and, moreover, we know its approximate size, location and shape as seen from this direction. We may still better locate it by placing a piece of paper cut exactly the size of the normal eye upon the plate between the four artificial landmarks, and mark up into the location of the foreign body. Then, in turn, we place the paper over the area between the landmarks upon the patient, and designate the location of the foreign body by means of an aniline pencil.

In the antero-posterior exposure two pieces of wire may be used. They are placed one upon the upper, the other upon the lower lid in such a manner as to represent the diameter of the eye from above downward. Comparisons are in like manner made.

In using the Haab magnet Dr. Appleby prefers the recumbent posture, as the patient is under better control. This is particularly important in cases in which the amount of traumatism is great.

Knowing the position of a foreign body lodged in the interior of the eye, we elect as to whether we shall remove it through the tract of entrance; draw it by means of a large magnet into the anterior chamber, and afterward through a corneal section remove it with the small point of the Hirschberg magnet; or we may decide that a meridional or an equatorial incision is preferable. In each instance it is our aim to remove the iron by such a method that it will result in the least injury to the eye.

The Haab magnet is much used as an important factor in diagnosis. When used for that purpose, the head of the patient is brought gradually toward the pole of the magnet. If pain be then present or increased, we know that the foreign body has impinged upon the tissues; finally, if after we have turned on the full force of the current, getting no pain nor increase of pain, we reverse the current, and apply the magnet to the eye so that its power-lines shall have had effect from all directions; then if the patient complains of no pain, we infer either that there is no foreign body of magnetic nature within the eye, or that it is too firmly imbedded to be moved by the magnet. In every case it is of course of the greatest importance to see the patient as soon after the injury as possible.—*Medical Review of Reviews.*

---

### TREATMENT OF ACUTE, ANTERIOR URETHRITIS.*

The treatment of this disease is often unsatisfactory because it is so difficult to find a drug which is beneficial in all cases. The abortive treatment is not recommended unless the case is seen very early, as any local application, which will abort it in two or three days, usually sets up a severe inflammation and injures the urethra. Some of the remedies used to abort the disease are carbolic acid (1 in 20), potassium permanganate (one per cent.), silver nitrate (20 grains to the ounce), protargol, argonin and peroxid of hydrogen. These have all been tried by the writer, and in the majority of cases have failed.

It is better in the early stage to treat on general principles, viz.: Rest in bed, if possible, or, in lieu of this, use a snugly fitting suspensory bandage; the penis should be kept very clean. The bowels should be relaxed by the use of small doses of calomel or

*By W. A. Hackett, M. B., M. C. P. S.

pill cathartic, and the patient advised to drink plenty of water. If he goes to bed, the diet should be reduced about one-half and should consist of milk, bread, eggs and light puddings. No vinegar, mustard, pepper, spiced food or alcohol in any form should be allowed. The use of tobacco, strong tea, coffee or cocoa should be prohibited, but lemonade and mineral waters may be given. All mental and sexual excitement must be avoided. The acidity of the urine may be reduced by potassium citrate or potassium acetate or liquor potassa. Heat favors the growth of the gonococci, hence the cold coil around the penis will have a good effect. If the patient be in bed he should not be kept very warm. He should be warned of the danger of infecting the eyes and the gravity of such an accident. The best dressing for the penis is absorbent gauze, a piece about four inches square with a slit cut in the center, through which the glans is passed until the gauze is well behind the corona. The foreskin is then drawn forward, thus causing the free end of the gauze to protrude beyond the preputial orifice. This dressing allows the discharge to drain freely from the meatus and keeps it from coming in contact with the prepuce and glans. If the prepuce be very short, the glans can be wrapped lightly in absorbent gauze. The dressing should be burned as soon as removed and the hands washed to prevent infection. The following is very useful in the early stages to render the urine antiseptic:

℞. Salol ........................................dr. i.
Boric acid........................................dr. i.
M. Div. in capsuli No. xii. Sig.—One four times a day.

This may be given from three to five days, but should be stopped as soon as the urine becomes dark or smoky.

To render the urine alkaline, alkaline water may be given freely, or one of the following:

℞. Potass bicarb ........................................ dr. 3.
Tinct. hyoscyam........................................dr. 2.
Aquae........................................add oz. 4.
M. Sig.—A tablespoonful in water after meals.

℞. Potass. acetat ........................................oz. 1.
Spts. ether, nitros ........................................dr. 2.
Syr. simp ........................................dr. 2.
Aquae........................................q. s. add oz. 3.
M. Sig.—A teaspoonful in water after meals.

For chordee and painful erections, the patient should empty the bladder before retiring and sleep on his side on a hard mattress, with light covering. When awakened by an erection, cold applications should be made to the penis. Cocain solution (1 per cent.) may be injected into the urethra and retained for several minutes. In severe cases, small doses of tincture opium may be given four or five times a day, or a large dose of sodium bromid or monobromid of camphor at bedtime. It is sometimes necessary to resort to suppositories of opium or morphine.

Injections as a rule should not be given at the very beginning when the symptoms are quite acute, except in conjunction with alkaline diuretics. The injections should be bland and non-irritating, and one which meets these requirements is gelatose silver or albargin. This is a light yellow powder and is entirely soluble in cold or warm water. Albargin is used in 0.1 to 0.2 per cent. solutions and is much cheaper than a number of the other new preparations.

The patient should be instructed in the use of the syringe, which should have a blunt conical tip and hold about 4 drams. After urinating, he gently inserts the nozzle of the filled syringe into the meatus, with lips pressed together against the syringe. The solution is thrown in slowly until a feeling of distention or discomfort occurs, when it is allowed to escape, or is kept in for a few minutes. This should be done three times a day. Hot boric acid solution may be used first. The following drugs are also useful in solution as injections, viz.: zinc sulphate, fl. ext. hydrastis, acetate of lead, protargol, argonin, potassium permanganate.

If the patient can come to the office every day, it is well to use a Valentine douche to flush out the anterior urethra, or a French soft catheter, No. 12, to which a syringe can be attached. During the latter part of the acute stage, capsules of copaiba and cubebs or methylene blue and yellow santal oil, five to ten drops in a capsule, one and a half hours after meals are very useful. In hospital practice the old Lafayette mixture is usually given. When the discharge becomes sticky in character it is well to stop the use of these remedies as they may delay the cure by over stimulation of the urethra, and give instead a mild alkaline diuretic. If the treatment has been successful, there is only a trifling urethral discharge in the morning, which soon disappears.

---

### SURGERY DURING THE WAR.

BY R. W. SIMPSON, JR.

Dr. J. Somers Buist, of Charleston, late surgeon Confederate States army, has prepared a statement of the wonderful vitality and pluck of General Rudolph Siegling, who was shot and left on the battlefield as dead, but who survived in a marvelous manner. The story from Dr. Buist was called out by the account of the terrible wound of Colonel R. Snowden Andrews. He says:

"Reading the account of Colonel Andrews' wound, and as our President's death has been caused by an abdominal wound, I would like to place on record the remarkable recovery of the late General Siegling, of Charleston, wounded at the battle of Second Manassas and then lieutenant in the German artillery.

"The battery in which Lieutenant Siegling commanded a section was ordered to take an advanced position. A battery of the Federals was directly opposite them. While advancing to the position a shell from a rifled cannon—known as a Hotchkiss shell—burst in front of the advancing company, wounding a corporal and taking off the cover of an ammunition chest.

"The base of the shell struck Lieutenant Siegling in the left groin, tearing away the clothing and sword belt and penetrating the abdominal walls. It made a rent about ten inches in length, exposing all the contents of the left abdominal regions, including the peritoneum and intestines. Shreds of clothing were carried into the wound, and, being exposed to soil and particles of vegetable matter, these also filled in the wound surfaces.

"Lieutenant Siegling by the force of the blow was hurled from his horse and fell in a trench between two corn hills, bleeding and in a semi-insensible condition. A man of strong nerve and great courage, and having in his early life taken a course of medical study, he had prepared himself for this very emergency, and taking the morphine in his possession at the time avoided the evil effects of the shock, which is so often the immediate cause of death on the battlefield.

"I reached him in about 15 minutes after the wounding and found him exhausted and in collapse from the loss of blood lying between the corn hills. The battle was still raging in our immediate front, and the whistling of the balls from the infantry as they passed above and around us could be heard and almost felt. The federal artillery were still in action, directing their fire on the very spot--shells exploding often so near as to cover us with earth.

"Under these circumstances hemorrhage was controlled, the wound dressed and, as soon as a stretcher could be obtained, the lieutenant was removed for a half mile along the firing line to the field infirmary in the immediate vicinity. An examination, in which I had the assistance of the most eminent surgeons of our command, convinced us that the injury was certainly mortal. The whole abdominal wall of the left side was open, the peritoneum torn and lacerated, the intestines not cut, but bruised and congested and exposed, being filled with dust, leaves and shreds of clothing. In those days we had no hypodermic syringe, nor did we have an idea of the antiseptic treatment of the wounds. Pure spring water was the best we could obtain, and this being freely used, the wound was closed after the abdominal contents had been returned and entered as far as the extreme laceration would permit.

"The lieutenant was made as comfortable as possible, notified of his condition, which he fully realized, and made all his preparations for death—even dictating his last will. So certain was the opinion of the fatal termination of his injuries that one of his fellow officers (Mr. Simons) had his coffin made from the boards of a fence in the neighborhood and the suit of uniform to bury him laid out. As it turned out, he had no use for it, and I was informed that the gallant Colonel Gadberry, of Newberry, was buried in this coffin.

"The treatment of this wound, partially by the closed and open method, was with a cold wet pack of pure spring water, just as in the case of Colonel Andrews.

"Lieutenant Siegling rallied both from the shock and the operation. He remained under my care for four days, and then being under orders from General Lee to rejoin the army then in Maryland I transported him, in the enfeebled condition in which he was, eight miles over a rough road in a rough ambulance, and left him under a tent-fly in the orchard attached to a country house under the care of a local physician. Lieutenant Siegling progressed favorably to recovery after many weeks of confinement to his bed. The wound healed by second intention, as it is surgically known.

"Lieutenant Siegling, after months of convalescence, made a complete recovery and returned to his command, and he served with distinction until the close of the war. Among surgeons of that day, of whom I may mention the late Dr. Darby and Dr. Taylor, of Columbia, as well as the leading men of the army, his recovery was recorded as one of the most remarkable of the time."
—*The Sunny South.*

---

### HYSTERICAL RELIGION AND SCIENCE.

A short time ago we were introduced, in the daily papers of Toronto, to the doings and sayings of the advocates of Divine Healing. It will not be amiss to pass under review a few of the remarkable statements made at the gathering held at Munro Park a few weeks ago. In what we shall say we are aiming no shaft at anything that is good or worthy in religious thought; nor do we desire, in the least, to say a single word against the teachings of the Nazarene.

But there is a limit to all things. When men and women, who have paid no special attention to disease, and who know practically nothing about pathology, undertake to descant upon these topics and the cure of disease, the public should be duly warned. It is an easy task for these people to shout about the wonderful cures they perform, but it is only reasonable to ask for proof. Their cases should be properly attested by competent physicians or surgeons before and after the so-called cure.

No doubt certain imaginary ailments have been cured in the past at the shrines of the old Greek and Roman gods, at the feet of the Egyptian sorcerers, and by the Indian medicine man who appealed to the virtue of some charm, or the Great Spirit on the top of the mountain, or to the white goat-skin nailed to the pine tree. From such circumstances and events grew up a belief in the efficacy of mysterious agencies in the cure of disease. What better could be expected in the days of Homer, when he speaks of the spleen as the seat of the mind and feelings, and as being surrounded by black bile?

One thing stands out as at once a refutation of the claims of Divine Healers: That we have never yet been furnished with proof of organic diseases being cured. If God is pleased to cure diseases by the methods of these healers, why is it he does not save the life of some devout and lovely Christian character who is ill of cancer or granular kidney? Surely it cannot be alleged that God has only the power to cure functional disorders, but falls short of that requisite for the organic affections! Further, it will not be contended by the most extreme Divine healers that the proper degree of faith is only found among those functionally afflicted, but that none of those who have had cancers, or tumors of the brain, or sarcoma of the bones, have been able to appeal with such faith as to gain the ear of the Creator. It is not denied that the Creator has power to cure a cancer, but he only chooses to do so when proper means are employed.

If a person has been genuinely cured by faith, on any one occasion, of a real disease, there can be no logical escape from the conclusion that he can be cured again and again. In this way it can be shown that with the proper balance of faith he can live on forever. Each time he becomes ill he is cured, and so on *ad infinitum*. But more. The same faith that can cure can avert. He need never be ill. Nay, more. The same faith that can prevent diseases and cure them can maintain perpetual health, perpetual youth in the tissues. They neither wear out nor die out. On their own ground these mistaken people are driven into the *reductio ad absurdum* of establishing God's law, man is mortal, as a fallacy!

Returning to the recent convention, one person was claimed to have been cured of consumption, but there was no proof of the bacilli being found. Another person had some trouble with his voice. We all know how often this is a mere form of hysteria, or passing nervousness, especially about the age of puberty, when the trouble began. A third case was that of a person who had a terrible eruption on her face. She was in a hospital for a time. In a short time she got well. What have we here? Does not every physician see the ear marks of some dermatitis that got well, and very likely largely through what was done for her in the hospital? And yet another had been cured of almost total deafness and threatened loss of voice. Hysterical deafness, blindness, aphonia, lameness, numbness, vomiting *et al.*, are not new. They were present in the siege of Troy, during the days of the Jewish captivity, and among the fakirs in the Buddhist temples. But, *mirabile dictu*, this is not all. A tooth was pulled and there was hemorrhage. The elders are called in. At this juncture the artery contracts and retracts and closes by a blood-clot, just as happens in the foot of a tom cat, or in the scalp of a Dyak of Borneo, who is not supposed to pray nor to call in the elders of his church. One other person had brain disease, heart disease, nervous debility and indigestion. A grand quadruple alliance, but such as at once declares its nature as not nearly so formidable as a little bit of tubercle in the supra-renal glands, and much more amenable to the influence of a fanciful mind over some fanciful symptoms that led the person to think she had four grave diseases.

When and where is all this wild ignorance to end? Is it not about time that persons who do not study diseases should cease preaching about them? Surely it is not too much to ask that those who are guided by sound methods of Biblical criticism should denounce all such visionary and wrongful applications of Holy Writ. If there is anything laid down in the New Testament with greater emphasis than another, it is to make due use of the means at our command.

What about the child that is too young to pray, or has not got praying parents? Or what is to be the fate of the man ill with pneumonia or typhoid fever, where there is too much stupor for him to place faith in any subject or person? Must the man who is rendered unconscious by a blow on his head receive no treatment because, in his helpless torpor, he is totally oblivious of his own existence, or the existence of anything else? A child is ill with diphtheria. Are the onlookers to pray or to send for a competent physician who can administer the antitoxine, or other proper remedies? These misguided people are guilty of a grave offense against society by propagating erroneous opinions.—*Canadian Practitioner and Review.*

---

### THE ANARCHIST AND THE SUBSTITUTOR.

The following from the *Texas Medical News* very well presents the growing evil of substitution :

In this great country of ours, where every man, woman and child are permitted to have as perfect freedom as possible, we are confronted with two very serious problems, namely: the suppression of anarchy, which must be done by means of an amendment to the Constitution of the United States, so as to make an attempt upon the life of a president or other high official who is filling a public trust by a choice of the people that of treason and punishable by death.

While speaking of this great evil, which has been brought so prominently and forcibly to our minds by the death of President McKinley, we cannot refrain referring to another evil in this country that is growing daily, and that is doing great damage to the medical profession, the legitimate manufacturing chemist and druggist, and even the people at large, and that is the evil of substitution as practiced by certain druggists in almost every town of any size.

Since we have devoted editorial space to a discussion of the first of the above evils elsewhere, we shall confine our remarks in this instance to the latter.

There is no doubt that when a physician prescribes for a patient by writing a prescription for the drug or combination of drugs which he thinks are most suitable for that particular case, he alone should be the judge of what is wanted. If his prescription calls for a certain manufacturer's ergot, or veratrum, or combination of drugs or preparatory preparations, it should be filled exactly as ordered, and it is to his interest and the interest of the patient to see that his orders are carried out in detail.

It seems to us that any physician of intelligence would take the time and trouble to examine the prescriptions which his patients have filled, and see that they get what he ordered and as designated, and when he finds a substitution has been made, then render a complaint to the druggist, and serve notice on him that if such methods are followed in the future his prescriptions will go elsewhere to be filled.

We have great respect for the druggist, and the relations existing between him and the physician are usually very close, and we regret to see the spirit of commercialism creeping into the methods of too many good, respectable appearing druggists.

Where radical measures are necessary to suppress this great evil they should be employed, and we are sure the upright, self-respecting, legitimate druggist will join the members of the profession in driving from their midst all such substitutors.

The reckless manner in which some houses prepare medicines and tablets and throw them upon the market, even the strongest poisons, with only one object---to undersell the legitimate manufacturer---is a menace to the people, and we are expecting to hear at any time of a very large shipment of A's tablets found to contain strychnine or morphine when they were labeled calomel, and as a result a wholesale killing follows.

There are many things we are careful about, but none more so than to know positively what medicine we take or give our families; where it was manufactured, and what druggist prepared it. The same rule should apply to the physician's patients, who look to him for protection.

---

### THE INCREASE OF RAPE IN THE SOUTH AND THE REASONS THEREFOR.

That rape is on the increase among the negroes of the South there seems to be no question, and this condition of affairs has developed *in toto* since slavery time.

For a negro to commit rape upon a white woman or child in the time of slavery was unheard of, not even during the four years of war, when the white men were away in battle and their families were at home unprotected and defenseless. The negroes of those days were not guilty of this most atrocious crime; but since the war, and particularly for the last decade, this crime has been on the increase.

Why is it, and what can be done to suppress such crimes? In the first place, we should consider the question of heredity, and count how many generations back to where we find the race one of savages in the jungles of Africa, and where the sexual act was performed *ad libitum* and by force, was not an uncommon thing. Here we find the passions strong and freely gratified. As the negro was brought to America and civilized, we find him under subjection, and placed under strict discipline and punished when rules were violated. He was, in most instances, well fed and clothed, and required to be regular in his habits and live an active outdoor life of labor. He was denied highly seasoned and indigestible foods, but greatest of all, his time was occupied by labor, and he did not have the time nor opportunity to allow his thoughts to run upon passionate lines.

What obtains now with the class who usually commit such crimes? They have grown up in an atmosphere of idleness and crime, taught the sexual act as soon as reaching the years of puberty, starving one season of the year and poorly clad and housed; again, in cotton-picking, feasting and gambling and drinking; but at all times, in thoughts and conversation, as well as indulgence, the sexual act is the most prominent idea in their nature, *or sexual intercoure is their highest ideal and the quintescence of perfect living.*

Is it any wonder that, under such circumstances, a race of moral degenerates is being developed—sexual perverts of the Kraft-Ebing type, and when such an individual, who possibly has not satisfied his sexual desire for some time, meets the opposite sex in an out-of-the-way place, unprotected and defenseless, his passions fire him on to satisfaction; he forgets everything else or the consequences that follow.

An enraged citizenship may well be excused for meting out justice by mob violence, either by hanging or even burning such criminals at the stake; but the example does little good as an object lesson for two reasons: Such criminals never read about such cases, nor do their fears control them when ruled by their passions.

Mob violence, however, seeks revenge, and really does prevent the further propagation of this degenerate species. How to prevent this crime is a most difficult problem. No doubt the education of the race, with educational instruction upon morals and right living, with proper religious teaching, is of inestimable value.

The encouragement of early marriages among the race is of great value, with due observance of the sacredness of the marriage vow, and above all the proper home circle, where the children are properly fed, clothed and reared, and have instilled into them wholesome thoughts, instead of being allowed to run about the streets and become saturated with crime.

Certain legislative enactments might be of some value, as well as precautionary measures on the part of the people not to ever allow an opportunity to arise permitting such a crime to be committed.

Such great leaders of the race as Booker T. Washington and Bishop Turner are doing good work towards stamping out this crime, only this work must be extended to the most lowly and squalid of the race.

My remarks, of course, are intended to apply only to the degenerate portion of the race, whose acts are as much condemned by the educated and refined negro as they are by the white race.

An intelligent discussion of this question, we think, is opportune and highly important at this time.—*Texas Med. News.*

---

### DISEASE ODORS.

Of the specific odors of disease two very marked cases come to mind: One, a young, buxom, red-cheeked woman, whose menstrual discharge was accompanied by such a pervasive odor that few could stay in the same room with her; the other, a man who suffered from profuse fetid perspiration confined to the axillary regions—the fluid could be seen constantly exuding, of a consistence a little heavier than normal perspiration, the disagreeable odor it yielded being very penetrating, so much so as to pervade the whole room and adhere to the furniture for hours after his departure.

The ammoniacal smell common to the aged, and due to retained or dribbling urine, is well known. Berard says that, apart from the excretions, an abnormal odor of the skin tends to draw flies, and that however little noticeable it may be it denotes death is near; and Boërhaäve held that a cadaveric odor always presages death. Althaus tells us that Skoda was hardly ever led into error by this indication,* and Compton also laid great stress upon this as a clinical symptom; but the smell given off during the "death agony" is totally different from the death odor (that of putridity) and is universally admitted to be specific.

The odors obtaining to sex are vastly different; thus in man it suggests mushrooms, in woman codfish.†

In gout the skin secretions take a special odor which Sydenham compared to whey; it is sour, or at least sourish, and there is an excess of ammonia. In rheumatism it is acetoformic, particularly in the regions of engorged articulations (Monin); it is a sour-smelling, acid perspiration.

In diabetes the smell is sweetish and mawkish, as of hay, according to Latham, "acetone," says Picot, and "midway between aldehyde and acetone, being due to a mixture in variable proportions of the two bodies," according to Bouchardat.

A musky odor obtains to several maladies, notably peritonitis, jaundice and icterus; and a stale, sour-beer odor to scrofulosis.

The pyemic person has a sweet, nauseating breath, with perhaps a flavor of new-mown hay.

In milk fever the smell is distinctly acid; in typhoid, musty, often with the odor of blood; in typhus, ammoniacal and mouse-like, which latter also obtains to faus; in intermittent the odor is that of fresh-baked brown bread; yellow fever has a cadaveric smell, or like the washings of a dirty gun-barrel.

In measles it closely resembles fresh-picked feathers; in diphtheria, is sickening and gangrenous—an odor that is absolutely

*[An odor of semen persisting about the body and apartment of an old man, even if he does not appear seriously ill, appears to be indicative of speedy dissolution. This is invariable, though why, except it is in a sense cadaveric, we are unable to explain. It most frequently obtains in connection with suppression of urine, and in the majority of cases points to prostatic disease of long standing.—ED.]

†[The odor of a perfectly healthy, cleanly woman should be that of thyme; the codfish odor is evidence of lack of personal cleanliness as regards the sexual organs, or of a diseased condition.—ED.]

pathognomonic; in smallpox, according to severity and stage, it ranges from that of the fallow deer to the dreadful one of the whole menagerie, or it may be that of burning horn or bones.

Hysteria usually develops an odor of violets or pineapples; sudamina, that of putrid straw; scabies, mouldy; anemia and cholera, ammoniacal (Drasch, Parker) and the discharges have either a semen or mush-room flavor.

Otorrhea has a peculiar, clinging, long-lasting odor that once observed will never be forgotten; so, too, is the odor of a hen-roost that obtains to ozenas and bad chronic catarrhs. Gangrene has an old, dead-meat smell, as have some cancers at certain stages—if there is much pus from an actively breaking-down, malignant growth, and especially in sarcomas, it is more like decaying fish.

At the onset of the plague the odor is sweet (Diemerboëck) or honey-like according to Doppner.

The atmosphere surrounding the chronic onanist will have a rotten mushroom-like odor, and an ill-kept libertine will combine this with a cod-fish smell.—*Clarke in Medical Recorder.*

---

### CHEAP JOHN SYPHILOGRAPHERS.

He who has had any opportunity of reading periodical medical literature has, beyond doubt, observed that many of the articles which appear have really no *raison d'être,* and are merely written for the purpose of affording their writers an opportunity of making themselves a little better known than those who cannot or dare not commit anything to paper. Among the writers there exist those who never can write anything but matter that is both interesting and instructive; and, on the other hand, there are those who are simply afflicted with a *cacoëthes scribendi,* whose every contribution is a bore and an infliction. It is not of those who merely rehash or condense more or less intelligently those things which have been well written and pass off the ideas of others as their own of whom we wish to speak, but rather of those who, with but a very vague idea of a subject, endeavor to create an impression that they know whereof they write, and make desperate attempts to speak *ex*

*cathedra* in a manner which simply makes their efforts appear ludicrous.

We are amused to see the desperate efforts which some medical writers make to elucidate some of the problems of syphilology. It is very often pitiful to look at the desperate efforts made by some to explain apparent obscurities which have long since been elucidated by acknowledged masters of the subject, who themselves hesitate to approach certain points which are decided off-hand by those who have no adequate knowledge of that concerning which they do not hesitate to express dogmatic opinions. We are continually confronted with ideas that are false, with others that are antiquated, and often with those that are distorted. We are occasionally greeted with an idea that has long been buried, and which we hoped had been relegated to the great collection of scientific fossils, and is resuscitated with no hope of survival by the writer, who possesses an enormous library on syphilology of six volumes, issued at the time when the quality of syphilis was still considered a burning question.

Were this all it might not be as serious a matter as the pseudo-syphilology with which we are presented in more pretentious works. Thus it is a matter of daily occurrence to find the subject of syphilis disposed of, in a work on general surgery, in some eight or ten pages. And in these few pages we find included ideas which are no longer accepted, views which are incorrect, and fallacies paraded which are certain to ultimately lead their reader into most disagreeable paths. The same may be said of the works on the principles and practice of medicine. These books do not as a rule take up what are now known as the specialties, but they will persist in giving a most incomplete idea of syphilis and its treatment. The disease is one which possesses a very large literature, which is constantly increasing, and when we speak of literature we mean those things written by authors of extensive experience, trained in observation, expert in pathology, and successful in treatment.

But what shall we say of those mainstays of medical students which but too often prove broken reeds to those who depend upon them—medical compends? Whilst good as reminders on general subjects, but very few are such in any of the specialties, and he who thinks that he can obtain a medical education from them shall certainly be disappointed. As a guide to more extensive quizzing they are useful, and as companions to the larger works they are deserving of support; but as the only books to be consulted they are deceptive, incomplete, and disappointing. And, unfortunately, it is on these very inadequate little books that many writers depend to round out their articles.

Large reading, much observation, experience of a clinical nature, and a due appreciation of the subject in hand will make a syphilographer. The mere fact of having read a few inadequate articles and a few old books will not entitle any one to claim a knowledge of one of the most complicated diseases with which we are called upon to cope. Had the majority of the medical profession but a slight idea of the immense literature of the subject, of the almost infinite complications which arise, of the difficulties of treatment in many cases, it would readily refrain from writing so much on the subject and appreciate more than ever the truth of the lines:

> "A little learning is a dangerous thing;
> Drink deep, or taste not the Pierian spring."

—*St. Louis Medical and Surgical Journal.*

---

### THE PROPAGATION OF YELLOW FEVER; OBSERVATIONS BASED ON RECENT RESEARCHES.*

Walter Reed gives the results of his own experience with regard to the manner in which yellow fever propagates itself. The infection of non-immune individuals was attempted in three ways, viz.: first, by the bites of mosquitoes that have previously bitten cases of yellow fever; secondly, by the injection of blood taken during the early stages from the general circulation of those suffering from the disease, and, thirdly, by exposure to the most intimate contact with fomites. The various observations are reported in detail. Under strict quarantine regulations they succeeded in conveying the disease to ten non-immunes by means of the bites of mosquitoes that had previously been fed on cases of yellow fever, at intervals varying from sixteen to fifty-seven days,

* N. Y. Med. Rec., Aug. 10, 1901.

before being applied to the person to be infected. The attack of yellow fever always followed the bite of the mosquito within the period of incubation of the disease. Three positive results were obtained by the subcutaneous injection of blood taken from the general circulation on the first and second days of the disease. The production of yellow fever in this way shows that the parasite is present in the general circulation, that the passage through the body of the mosquito is not absolutely essential in the life history of the micro-organism, and that the period of incubation of the disease, when thus produced, corresponds fairly closely to that occasioned by the mosquito bite. All attempts to propagate the disease by fomites were entirely negative. In no case was the disease produced by contact with fomites. Seven non-immunes slept in the bed and garments just vacated by cases of yellow fever, remained in a building to which no sunlight ever came, and in which the circulation of air was purposely made as defective as possible, engaged in packing boxes with garments much soiled by contact with the bodies and excreta of yellow fever patients, and not one of them contracted the disease. It is not believed that the enforcement of the most rigid hygienic regulations, such as we have hitherto known them, will prevent the propagation of this disease, provided it should again be imported into this country. It can no longer be classed with the "filth diseases." In the light of the newer observations, the writer affirms that we have to deal with the same source of infection to which we now trace the malarial fevers, the mosquito, substituting culex for anopheles.—*St. Louis Med. Record.*

---

### ROENTGEN SOCIETY.

The Roentgen Society of the United States will hold its second annual meeting in Buffalo in the University building, September 10 and 11, 1901, under the presidency of Dr. Heber Robarts, of St. Louis. The secretary is Dr. J. Rudis Jicinsky, of Cedar Rapids, Iowa.

